# Efficient Plant Regeneration System from Leaf Explant Cultures of *Daphne genkwa* via Somatic Embryogenesis

**DOI:** 10.3390/plants12112175

**Published:** 2023-05-30

**Authors:** Seong Sub Ku, Hyun-A Woo, Min Jun Shin, Eun Yee Jie, HyeRan Kim, Hyun-Soon Kim, Hye Sun Cho, Won-Joong Jeong, Moon-Soon Lee, Sung Ran Min, Suk Weon Kim

**Affiliations:** 1 Plant Systems Engineering Research Center, Korea Research Institute of Bioscience and Biotechnology, Daejeon 34141, Republic of Korea; rntjdtjq@kribb.re.kr (S.S.K.); kimhr@kribb.re.kr (H.K.); hyuns@kribb.re.kr (H.-S.K.);; 2 Department of Industrial Plant Science and Technology, Chungbuk National University, Cheongju 28644, Republic of Korea; 3 Biological Resource Center, Korea Research Institute of Bioscience and Biotechnology, Jeongeup 56212, Republic of Korea

**Keywords:** auxin, embryogenic structure, medicinal plant, somatic embryo

## Abstract

This study aimed to establish an efficient plant regeneration system from leaf-derived embryogenic structure cultures of *Daphne genkwa*. To induce embryogenic structures, fully expanded leaf explants of *D. genkwa* were cultured on Murashige and Skoog (MS) medium supplemented with 0, 0.1, 0.5, 1, 2, and 5 mg·L^−1^ 2,4-dichlorophenoxyacetic acid (2,4-D), respectively. After 8 weeks of incubation, the highest frequency of embryogenic structure formation reached 100% when the leaf explants were cultivated on MS medium supplemented with 0.1 to 1 mg·L^−1^ 2,4-D. At higher concentrations of 2,4-D (over 2 mg·L^−1^ 2,4-D), the frequency of embryogenic structure formation significantly declined. Similar to 2,4-D, indole butyric acid (IBA) and α-naphthaleneacetic acid (NAA) treatments were also able to form embryogenic structures. However, the frequency of embryogenic structure formation was lower than that of 2,4-D. In particular, the yellow embryonic structure (YES) and white embryonic structure (WES) were simultaneously developed from the leaf explants of *D. genkwa* on culture medium containing 2,4-D, IBA, and NAA, respectively. Embryogenic calluses (ECs) were formed from the YES after subsequent rounds of subculture on MS medium supplemented with 1 mg·L^−1^ 2,4-D. To regenerate whole plants, the embryogenic callus (EC) and the two embryogenic structures (YES and WES) were transferred onto MS medium supplemented with 0.1 mg·L^−1^ 6-benzyl aminopurine (BA). The YES had the highest plant regeneration potential via somatic embryo and shoot development compared to the EC and WES. To our knowledge, this is the first successful report of a plant regeneration system via the somatic embryogenesis of *D. genkwa*. Thus, the embryogenic structures and plant regeneration system of *D. genkwa* could be applied to mass proliferation and genetic modification for pharmaceutical metabolite production in *D. genkwa*.

## 1. Introduction

The genus *Daphne* belongs to the Thymelaeaceae family and contains over 90 species that are distributed throughout Asia, Europe, and North Africa [1]. In particular, *Daphne genkwa* Sieb.et Zucc. Is a well-known medicinal shrub plant in China and Korea [2]. The phytochemical composition of the genus *Daphne* is quite diverse and it produces several classes of secondary metabolites including coumarins, flavonoids, lignans, terpenes and other alkaloids [2]. Especially, the flower buds of *D. genkwa* have been used for diuretic, antitussive, anticancer, and abortifacient purposes [3,4,5]. Flavonoid fractions extracted from the flower buds of *D. genkwa* exhibit anti-inflammatory, analgesic and immunomodulatory activities [3,6]. Thus, the plant species belonging to the genus *Daphne* can be considered an important biological resource both for the treatment of various diseases and as possible leads in the discovery of new medications [2].

*Daphne* species are very popular because of their delightful, fragrant and attractive flowers [7]. The commercial propagation of *Daphne* is typically from seed or vegetative cutting [8]. However, there are several problems associated with the propagation of *Daphne*. Seed propagation often produces non-uniform materials because of genetic segregation. Moreover, *Daphne* has been categorized as a ‘‘difficult-to-root’’ group, both with conventional propagation via cuttings and following micropropagation [9]. Furthermore, one of the major limitations to *Daphne*’s cultivation is susceptibility to fungal root pathogens [10] and viruses [11]. These limitations could be overcome by the multiplication of disease-free plants through plant tissue culture technology [8].

Micropropagation may be a good alternative method to produce ornamental and medicinal shrubs [12]. However, there have not been many studies on the in vitro mass propagation of *Daphne* species. An in vitro propagation system of several *Daphne* species was established from nodal explants by shoot multiplication using cytokinins such as BA, 2iP and TDZ [8,13]. Wiszniewska et al. [14] also reported that three *Daphne* species were propagated using media enriched with natural ingredients including coconut water and pineapple pulp for rooting. Nowakowska et al. [15] achieved the 100% shoot regeneration of *D. mezereum* cv. Alba. They insisted that the type and concentration of plant growth regulators had an essential effect on the regeneration and growth of the shoots of *D. mezereum* cv. Alba in the in vitro culture. Recently, Nowakowska and Pacholczak [16] reported that meta-topolin had a more positive effect on shoot regeneration from explants of *Daphne mezereum*. Although studies related to in vitro shoot multiplication from nodal cultures of *Daphne* species have been reported, no study has yet reported successful plant regeneration through the somatic embryogenesis of *Daphne* species.

Therefore, this study aimed to establish an efficient plant regeneration system through the somatic embryogenesis of *D. genkwa*. To achieve this goal, the effect of several auxin (2,4-D, IBA, and NAA) concentrations and types of culture media on the formation of embryogenic structures and the development of somatic embryos from leaf explants were examined. In addition, we investigated the effects of cytokinin and light requirements on the process of plant regeneration through shoot development from embryogenic calluses or embryonic structures.

## 2. Results

### 2.1. Plant Regeneration from Leaf-Derived Embryogenic Structures of D. genkwa

**Figure 1:**
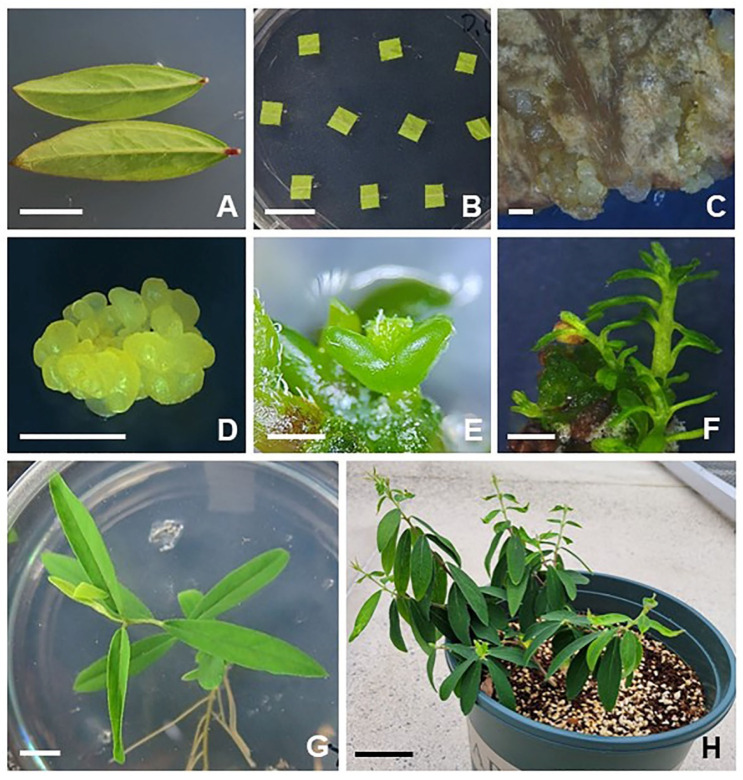
Somatic embryogenesis and plant regeneration from leaf explant cultures of *D. genkwa* cultured on Murashige and Skoog medium containing 2,4-D. (**A**) Fully expanded young leaves; (**B**) leaf explants; (**C**) initiation of embryogenic structure formation from leaf explants; (**D**) globular-shaped embryogenic structure; (**E**) heart- and torpedo-shaped somatic embryo; (**F**) shoot conversion and development of plantlets from somatic embryos; (**G**) rooting of plantlet; (**H**) regenerated plant transplanted to potting soil. Scale bars represent: (**A**,**B**,**G**) 1 cm; (**C**–**E**) 1 mm; (**F**) 2 mm; (**H**) 10 cm.

An efficient plant regeneration system from leaf-derived embryogenic structures of *Daphne genkwa* was established in this study (Figure 1). Fully expanded young leaves were used for embryogenic structure induction (Figure 1A,B). Yellow embryogenic structures (YESs) and small globular structures begun to form on the cut edge and leaf vein ends (Figure 1C) when they were cultured on Murashige and Skoog (MS) [17] medium supplemented with low concentrations of 2,4-D after 8 weeks of incubation (Figure 1C). When the yellow embryogenic structures were carefully transferred to fresh MS medium supplemented with 1 mg·L^−1^ 2,4-D (MS1D), they proliferated well (Figure 1D). In particular, embryogenic calluses (ECs) were formed from the YESs after subsequent rounds of subculture on MS medium supplemented 1 mg·L^−1^ 2,4-D. To induce somatic embryos, yellow nodular calluses were transferred to MS basal solid medium without growth regulators in the light. Yellow nodular calluses developed into globular-stage somatic embryos after 4 weeks of incubation. The globular-stage somatic embryos further developed into heart- and torpedo-stage embryos (Figure 1E). After a further 4 weeks of incubation in the light, the somatic embryos were successfully converted into shoots (Figure 1F). For root induction, the regenerated shoots were carefully cut into single stems and transferred to 1/2 MS with 0.1 mg·L^−1^ IBA. Roots were spontaneously formed at the ends of the elongated shoots at a low concentration of IBA treatment (Figure 1G). The rooted plantlet was successfully transferred into potting soil and maintained in a growth chamber (Figure 1H).

These results clearly showed that the initial yellow nodular structures developed from leaf explants had embryogenic potential. To our knowledge, this study is the first successful report of plant regeneration from the leaf explants of *D. genkwa* via somatic embryogenesis. Interestingly, other types of auxin treatments (IBA and NAA) were also able to regenerate whole plants via somatic embryogenesis from the leaf explants of *D. genkwa* (see Supplementary Materials, Figure S1). Similar to 2,4-D treatments, small globular structures began to form on the cut edge of leaf explants when cultured on MS medium supplemented with IBA (see Supplementary Materials, Figure S1B). The globular structures developed into heart-stage embryos when they were transferred to MS basal medium (see Supplementary Materials, Figure S1C,D). After a further 4 weeks of incubation in the light, somatic embryos were successfully converted into torpedo- and cotyledonary-stage embryos (see Supplementary Materials, Figure S1E,F), and these somatic embryos successfully developed into normal plants in the same way as for the 2,4-D treatment. Furthermore, the overall process of somatic embryogenesis from leaf explants by NAA treatment was almost similar to that of the IBA treatments. These results clearly showed that auxins played a stimulating role in the somatic embryogenesis from the leaf explants of *D. genkwa*.

### 2.2. 2,4-D Promotes Embryogenic Structure Formation from Leaf Explants of D. genkwa

**Figure 2:**
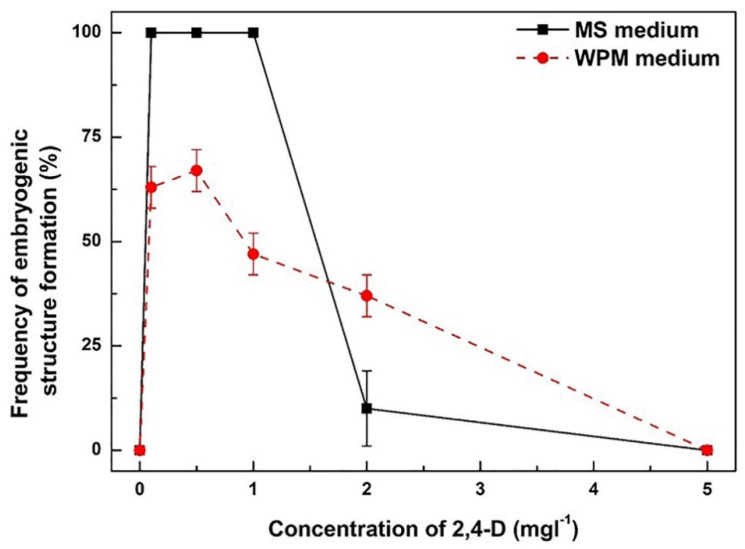
Effects of 2,4-D concentrations on embryogenic structure formation from leaf explant cultures of *D. genkwa* cultured on MS and WPM medium. Each symbol (–■–, –●–) represents the results after 8 weeks of culture in dark conditions. Results are expressed as mean ± SD (*n* = 3).

To examine the effect of 2,4-D concentration and culture medium types on embryogenic structure formation from leaf explants, leaf segments were cultured on MS and Lloyd and McCown’s woody plant medium (WPM) [18] supplemented with 0, 0.1, 0.5, 1, 2, and 5 mg·L^−1^ 2,4-D (Figure 2). After 8 weeks of culture, the highest frequency of embryogenic structure formation reached 100% when the leaf explants were incubated on MS medium supplemented with 0.1 to 1 mg·L^−1^ 2,4-D in the dark. However, the frequency of embryogenic structure formation significantly declined at the higher concentration of 2,4-D treatment (over 2 mg·L^−1^ 2,4-D). The leaf explants did not form any embryogenic structures at 5 mg·L^−1^ 2,4-D, even with the prolonged incubation period. Similar to the MS medium, leaf explants were able to form embryogenic structures when they were cultured on WPM medium supplemented with 0.1 to 1 mg·L^−1^ 2,4-D in the dark (Figure 2). The frequency of embryogenic structure formation from 2,4-D treatments was 0, 63, 67, 47, 37 and 0%, respectively. In the case of the WPM medium, the highest frequency of embryogenic structure formation reached 67% when the leaf explants were incubated on WPM medium supplemented with 0.5 mg·L^−1^ 2,4-D after 8 weeks of incubation. Similar to the MS medium, the frequency of embryogenic structure formation declined as the 2,4-D concentration increased. Furthermore, no embryogenic structures formed at the concentration of 5 mg·L^−1^ 2,4-D in the WPM medium. Compared to the MS medium, the frequency of embryogenic structure formation from the WPM medium was 0.7-fold lower than that of the MS medium at the lower concentration of 2,4-D treatment. Although the efficiency of embryogenic structure formation was higher with the MS medium compared to the WPM medium, there were no differences observed in the morphological characteristics of somatic embryo formation or plant regeneration between the two media.

### 2.3. Both IBA and NAA Promote Embryogenic Structure Formation from Leaf Explants of D. genkwa

**Figure 3:**
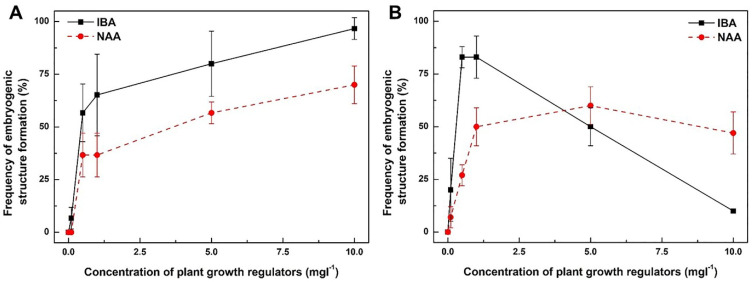
Effects of IBA and NAA concentrations on embryogenic structure formation from leaf explant cultures of *D. genkwa* cultured on MS (**A**) and WPM (**B**) media. Each symbol (–■–, –●–) represents IBA and NAA concentrations after 8 weeks of culture in dark conditions. Results are expressed as mean ± SD (*n* = 3).

The effect of different concentrations of IBA and NAA on embryogenic structure formation from leaf explant cultures of *D. genkwa* was examined (Figure 3). Although both IBA and NAA belong to the same kind of plant growth regulators as auxins, their overall effect on embryogenic callus formation was significantly different from that of 2,4-D (Figure 3). After 8 weeks of culture, the frequency of embryogenic structure formation from IBA treatments was 0, 7, 57, 65, 80 and 97%, respectively. The highest frequency of embryogenic structure formation reached 97% when the leaf explants were incubated on MS medium supplemented with 10 mg·L^−1^ IBA in the dark (Figure 3A). Interestingly, the frequency of embryogenic structure formation increased as the IBA concentration increased. In the case of the NAA treatments, the frequency of embryogenic structure formation was 0, 0, 37, 37, 57 and 70%, respectively. The highest frequency of embryogenic structure formation reached 70% when the leaf explants were incubated on MS medium supplemented with 10 mg·L^−1^ NAA in the dark (Figure 3A). Similar to the IBA treatments, the frequency of embryogenic structure formation from the NAA treatments increased as the NAA concentration increased.

The effect of IBA and NAA on embryogenic structure formation from leaf explants was also examined using WPM medium instead of MS medium (Figure 3B). The effect of the lower-concentration treatments (both IBA and NAA) on embryogenic structure formation on WPM medium was similar to that on MS medium, but the frequency of embryogenic structure formation on WPM medium with a concentration over 5 mg·L^−1^ was reduced, even if the IBA and NAA concentration increased. Overall, the frequency of embryogenic structure formation on WPM medium was slightly lower than that on MS medium. In particular, the frequency of embryogenic structure formation on WPM medium containing 0.5 to 1.0 mg·L^−1^ IBA was 1.3- to 1.5-fold higher than that on MS medium.

In this study, we also investigated whether the effect of auxins (2,4-D, IBA and NAA) on embryogenic structure formation from leaf explants was affected in the presence of light (see Supplementary Materials, Figure S2). In the presence of light, the effect of embryogenic structure formation of all three auxins (2,4-D, IBA and NAA) significantly reduced regardless of the types and concentrations of the auxins and the types of culture media. In particular, the effect of 2,4-D was significantly different in the presence of light compared to dark incubation. Except for the 1.0 mg·L^−1^ 2,4-D treatment on MS and WPM medium, there was no embryogenic structure formation in any of the 2,4-D treatments (see Supplementary Materials, Figure S2). Even in the IBA and NAA treatments, the efficiency of embryogenic structure formation decreased by more than 3-fold compared to dark incubation. These results clearly showed that the presence of light had a negative role in somatic embryogenesis from the leaf explant cultures of *D. genkwa*.

### 2.4. BA Promotes Somatic Embryo Development from Embryogenic Callus and of Embryogenic Structures of D. genkwa

**Figure 4:**
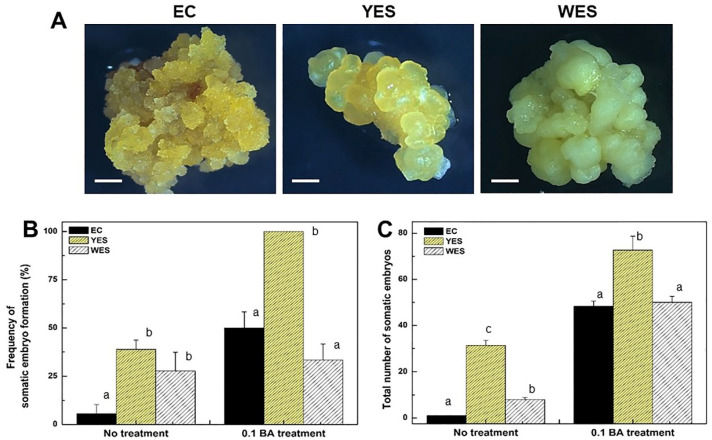
Effects of BA concentrations on somatic embryo formation of embryogenic callus (EC), yellow embryogenic structure (YES), and white embryogenic structure (WES) derived from leaf explant cultures of *D. genkwa*. (**A**) Different types of embryogenic structures; (**B**) frequency of somatic embryo formation and (**C**) the total number of somatic embryos on MS medium with or without 0.1 mg·L^−1^ BA after 4 weeks of incubation in the light. Results are expressed as mean ± SD (*n* = 3). Different lowercase letters on the bars indicate significant differences between each treatment (*p* < 0.05).

Yellow embryogenic structures (YESs) were simultaneously formed from the leaf explants of *D. genkwa* on culture medium containing 2,4-D, IBA, and NAA, and white embryogenic structures (WESs) were formed on culture medium containing IBA and NAA. When the YES was subcultured twice in MS1D medium, embryogenic calluses (EC) were formed (Figure 4A). To investigate whether the embryogenic callus and the two embryogenic structures from leaf explants had different plant regeneration potentials, the EC, YES, and WES were transferred onto MS medium supplemented with or without 0.1 mg·L^−1^ BA. After 4 weeks of incubation in the light, the frequency of somatic embryo formation and total number of somatic embryos were determined (Figure 4B,C). The frequency of somatic embryo formation from the EC, YES, and WES was 5.5, 38.9, and 27.8%, respectively, when cultured on MS basal medium. However, the frequency was significantly increased to 50, 100 and 33.3% when they were cultured on MS medium supplemented with 0.1 mg·L^−1^ BA (Figure 4B). Except for the WES, the frequency of somatic embryo formation greatly increased in the presence of low concentrations of BA. Additionally, the total numbers of somatic embryos from the EC, YES, and WES were 1, 31.3, and 5, respectively, when they were cultured on MS basal medium.

However, the total numbers of somatic embryos significantly increased to 48.3, 72.7 and 50, respectively, when cultured on MS medium supplemented with 0.1 mg·L^−1^ BA (Figure 4C). These results clearly showed that low concentrations of BA had a stimulating role in the development of somatic embryos from the initial embryonic structures of *D. genkwa*. In addition, it showed that the YES had greater embryogenic potential to differentiate into somatic embryos than the EC and WES.

### 2.5. Conversion of Somatic Embryos into Plantlets

**Figure 5:**
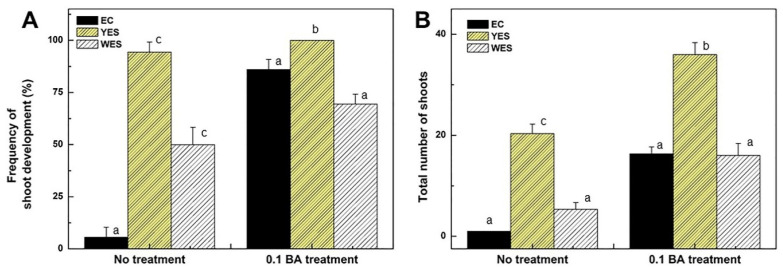
Effects of BA concentrations on frequency of shoot development and number of shoots of embryogenic callus (EC), yellow embryogenic structure (YES), and white embryogenic structure (WES) derived from leaf explant cultures of *D. genkwa*. (**A**) Frequency of shoot development and (**B**) total number of shoots on MS medium with or without 0.1 mg·L^−1^ BA after 8 weeks of incubation in the light. Results are expressed as mean ± SD (*n* = 3). Different lowercase letters on the bars indicate significant differences between each treatment (*p* < 0.05).

After 8 weeks of incubation in the light, the frequency of shoot development and the total number of shoots were examined (Figure 5). The frequency of shoot development from the EC, YES, and WES was 5.5, 94.4, and 50%, respectively, when cultured on MS basal medium. However, the frequency significantly increased to 86.1, 100 and 69.4%, respectively, when cultured on MS medium supplemented with 0.1 mg·L^−1^ BA (Figure 5A). In the case of the EC, somatic embryos derived from the EC did not fully develop into shoots on MS basal medium. However, the efficiency of shoot development from somatic embryos significantly increased when 0.1 mg·L^−1^ BA was supplemented. In the YES and WES, the efficiency of shoot development from somatic embryos increased by 1.1- to 1.4-fold when 0.1 mg·L^−1^ BA was supplemented. Additionally, the total numbers of elongated shoots from the EC, YES, and WES were 1, 20.3, and 5.3, respectively, when cultured on MS basal medium. However, the total numbers of shoots significantly increased to 16.3, 36 and 16, respectively, when cultured on MS medium supplemented with 0.1 mg·L^−1^ BA (Figure 5B). These results clearly showed that low concentrations of BA had a stimulating role in shoot differentiation from the somatic embryos of *D. genkwa*. Furthermore, it showed that the YES is a more optimal tissue for plant regeneration than the EC and WES.

To regenerate whole plants from shoots, regenerated shoots were transferred to 1/2 MS medium supplemented with 0.1 mg·L^−1^ IBA. After 4 weeks of incubation in the light, approximately 20% of them were successfully rooted. In this study, the total period required for plant regeneration from the leaf explants of *D. genkwa* was about 30 weeks; callus induction from leaf explant: 8 weeks; callus proliferation: 6 weeks; somatic embryo formation from callus: 4 weeks; plantlet conversion from somatic embryos: 4 weeks; rooting and acclimatization: 8 weeks. The plant regeneration system established in this study is expected to be used for in vitro mass propagation or quality improvement studies of *D. genkwa*.

## 3. Discussion

The efficient plant regeneration system from leaf-derived embryogenic structure cultures of *Daphne genkwa* was established in this study (Figure 1). To our knowledge, this is the first successful report of a plant regeneration system via the somatic embryogenesis of *D. genkwa*. To date, in vitro propagation systems of several *Daphne* species have been established from nodal explants by shoot multiplication using cytokinins [8,13,16]. Furthermore, adventitious shoot formation and somatic embryogenesis from leaf explants has not been reported yet. However, this study showed that plant regeneration is possible via somatic embryogenesis from the leaf explants of *Daphne* species. Although shoot proliferation methods using adventitious shoots and axillary buds have been widely applied to in vitro propagation, plant regeneration via somatic embryogenesis may offer many advantages over organogenesis, such as the feasibility of a single-cell origin and the possibility of automating the large-scale production of embryos in bioreactors [19]. However, the application of somatic embryogenesis in a wide range of woody plants is limited by genotypic influences, poor germination of somatic embryos, and limited numbers of explants [20]. Even with these limitations, somatic embryogenesis has the potential to produce plants through in vitro propagation, and has now become a routine protocol for many trees [21]. Somatic embryogenesis can be induced in vitro from a range of plant explant types, such as embryogenic callus tissues, cotyledons, and zygotic embryos [22]. In woody plants, an embryogenic callus was initiated from immature or mature zygotic embryos as initial explants [20]. In general, immature tissues and organs more easily produce somatic embryos compared with old or adult plant explants [23]. Thus, immature embryos have been the most frequently used explants in somatic embryogenesis [24]. However, many studies have already been reported that plant regeneration is possible through somatic embryogenesis from leaf explants of species such as *Phalaenopsis* [25], peace lily [26], *Citrullus colocynthis* [27], *Capsicum baccatum* [28], *Tolumnia* species [29], *Scaevola sericea* [30], and *Euryodendron excelsum* [31]. In this study, embryogenic cells were successfully induced from the leaf explants of *D. genkwa*. It is expected that the plant regeneration system established in this study could be applied to other *Daphne* species for in vitro propagation.

To establish the in vitro proliferation system in plant tissue culture, nutrient medium and plant growth regulators are one of the most important factors. In general, WPM medium is frequently used for woody plants, whereas MS medium is often applied in herbaceous plants [32]. In this study, we found that MS medium was more suitable for somatic embryogenesis from the leaf explants of *D. genkwa* than WPM medium (Figure 2 and Figure 3). Recently, Zhang et al. (2023) reported that WPM medium was more suitable for whole plants from immature seeds of *Akebia trifoliata* via direct somatic embryogenesis [33]. Nowakowska et al. (2019) also reported that WPM medium was suitable for shoot regeneration in *D. mezereum* ‘Alba’, especially in long-term cultures, probably due to a lower content of macro-elements resulting in a slower maturing and aging of explants [15]. However, Noshad et al. (2009) determined the suitable medium for nodal cultures for shoot proliferation from seven *Daphne* species [8]. Five of the species responded best on MS media, while the remaining two species performed best on WPM media. These previous reports show that the optimal medium conditions are different for each plant species. In this study, the regenerated plants from leaf-derived somatic embryos of *D. genkwa* did not show severe death in MS medium.

To study somatic embryogenesis, the use of a defined medium and a single-step transfer of a callus growing in a medium supplemented with a moderate dose of 2,4-D to one containing a reduced amount of the auxin or none at all was adopted as the standard protocol for inducing somatic embryogenesis in a broad range of species [34,35]. In this study, the somatic embryogenesis from leaf explants was induced, with the best results being on MS medium containing 0.1 to 1 mg·L^−1^ 2,4-D (Figure 2). Both IBA and NAA could also induce somatic embryogenesis from leaf explants, although the efficiency was slightly lower than that of 2,4-D (Figure 3). In preliminary studies, we also examined the effect of a cytokinin (benzyl aminopurine) on somatic embryogenesis from leaf explants (see Supplementary Materials, Figure S3). No embryogenic structure formation was observed in any of the treatments with various BA concentrations regardless of medium type. Only a direct shoot was formed, and only in the 0.1 mg·L^−1^ BA treatment. These results clearly show that somatic embryogenesis can be easily induced from the leaf explants of *D. genkwa* by auxins only. The auxin treatment of explants was reported to be an indispensable inducer of somatic embryogenesis in a large number of plant species [36,37]. Treating tissue cultured in vitro with auxins results in the extensive reprogramming of the somatic cell transcriptome, which involves the modulation of numerous somatic-embryogenesis-associated transcription factor genes [37,38,39]. However, different auxin types can generate different physiological responses during somatic embryogenesis in spruce species [40]. Hazubska-Przybył et al. [40] reported that the quality of the germinated embryos of *P. abies* and their development into plantlets depended on the auxin type and were the highest in NAA-originated embryos. In this study, we also confirmed that embryogenic structure formation, somatic embryo development, and their plantlet regeneration depended on the auxin type and were the highest in 2,4-D-induced embryos.

In the presence of light, the efficiency of embryogenic structure formation decreased by more than 3-fold compared to the dark incubation regardless of the types and concentrations of auxins (see Supplementary Materials, Figure S2). These results clearly showed that the presence of light had a negative role in somatic embryogenesis from the leaf explant cultures of *D. genkwa*. This result is consistent with the report that light has an inhibitory effect on the somatic embryogenesis of carrot [41]. Furthermore, Kintzios and Taravira [42] reported that the intensity of light significantly affected the rate of somatic embryogenesis, embryo maturation, and plant regeneration. Recently, the requirement of light for somatic embryogenesis has been documented in many species; however, its exact mechanism has not been fully elucidated [43].

Interestingly, yellow embryogenic structures (YES) were simultaneously formed from the leaf explants of *D. genkwa* on culture medium containing 2,4-D, IBA, and NAA, and white embryogenic structures (WES) were formed on culture medium containing IBA and NAA. When the YES was subcultured twice more in MS1D medium, embryogenic calluses (EC) were formed (Figure 4A). The frequency of somatic embryo formation from the YES was higher than that of EC and WES regardless of the supplementation of BA in this study. Furthermore, the frequency of shoot development from somatic embryos was also highest in the YES (Figure 5). These results clearly show that the YES has better plant regeneration potential than the other types of embryogenic calluses of *D. genkwa*. Armstrong et al. (1985) classified maize calluses into three types, namely, I-, II-, and III-type calluses, based on the callus characteristics. Among these types, only the II-type callus, known as the embryonic callus, has cell totipotency and the ability to regenerate into whole plants [44]. The difference in plant regeneration potential of four different types of calluses of *Miscanthus* x *giganteus* including the shoot-forming callus, embryogenic-like callus, friable callus and yellow root-forming callus was also reported [45]. The loss of plant regeneration potential with the long-term subculture of the callus is a critical limitation for plant regeneration and the mass proliferation of useful medicinal plants, which has been observed for other plant species [45,46,47]. In a transcriptome analysis of embryonic calluses from maize with strong redifferentiation capacity and with weak redifferentiation capability, the upregulation of WOX genes promoted plant regeneration from embryogenic calluses in high-regeneration maize lines [48]. Thus, the fine selection and maintenance of an embryogenic nodular callus type seems to be an important factor affecting the regeneration efficiency of embryogenic-like callus cultures. In this study, we confirmed that the regeneration competence of the YES of *D. genkwa* could be maintained for long-term culture periods (up to 1 year) by periodic subculture.

## 4. Materials and Methods

### 4.1. Plant Materials and Surface Sterilization of Leaf Explants

Mature plants of *Daphne genkwa* Sieb. et Zucc. were purchased from a botanical garden in Kyung-gi province in Republic of Korea. A voucher specimen (accession number KRIB 0093099) was preserved at the herbarium of the Korea Research Institute of Bioscience and Biotechnology.

Fully expanded young leaves of *D. genkwa* were used for the experiments. The leaves were collected and surface-sterilized in 70% alcohol for 1 min, and then soaked in a sodium hypochlorite solution containing 0.8% active chloride for 15 min with occasional agitation, and then washed five times with sterilized distilled water. Remaining moisture on the leaves was removed with sterile filter papers (Advantec, 70 mm) in a laminar hood.

### 4.2. Effect of Auxins (2,4-D, IBA, NAA) and Type of Culture Media on Embryogenic Structure Formation from Leaf Explants of D. genkwa

After surface sterilization, leaf explants were cut into small segments (approximately 5 mm^2^ in area) in a laminar hood. The leaf segments were placed onto callus-inducing medium. In this study, we used two types of culture medium for embryogenic structure induction. One was MS [17] medium and the other one was WPM [18] medium. The MS medium consisted of full-strength MS inorganic salts, 0.1 mg·L^−1^ thiamine·HCl, 0.5 mg·L^−1^ pyridoxine·HCl, 0.5 mg·L^−1^ nicotinic acid, 2 mg·L^−1^ glycine, 100 mg·L^−1^ myo-inositol, 30 g·L^−1^ sucrose and 4 g·L^−1^ Gelrite. The WPM medium consisted of full-strength WPM inorganic salts, 1 mg·L^−1^ thiamine·HCl, 0.5 mg·L^−1^ pyridoxine·HCl, 0.5 mg·L^−1^ nicotinic acid, 2 mg·L^−1^ glycine, 100 mg·L^−1^ myo-inositol, 30 g·L^−1^ sucrose and 4 g·L^−1^ Gelrite. The pH of all media was adjusted to 5.8 with 1 N NaOH before autoclaving.

To examine the effect of 2,4-D on embryogenic structure formation, leaf segments were placed onto MS and WPM medium supplemented with 0, 0.1, 0.5, 1, 2, and 5 mg·L^−1^ 2,4-D, respectively. The cultures were maintained at 25 °C in the dark or light (approximately 45 μmol m^−2^ s^−1^ from cool-white, fluorescent lamps with a 16 h photoperiod). Each treatment consisted of 10 explants in a plastic Petri dish (90 mm × 15 mm) and was repeated three times. After 8 weeks of culture, the frequency of embryogenic structure formation from leaf explants was determined. The embryogenic structures formed on leaf explants were transferred onto MS or WPM medium containing 1 mg·L^−1^ 2,4-D and maintained at 25 °C in the dark. The subculture was conducted at 4-week intervals. Similar to 2,4-D, the effect of IBA and NAA treatments on embryogenic structure formation from leaf explants was examined in the same manner. Leaf segments were transferred onto MS and WPM medium supplemented with 0, 0.1, 0.5, 1, 5, and 10 mg·L^−1^ IBA or NAA, respectively. All IBA and NAA treatments were prepared and cultured in the same way as the 2,4-D treatment. As mentioned above, the frequency of embryogenic structure formation from leaf explants was examined after 8 weeks of incubation. Subculture was carried out in the same manner as mentioned above.

### 4.3. Effect of Cytokinin on Development of Somatic Embryos and Shoot Differentiation from Embryogenic Callus and Embryonic Structures of D. genkwa

In preliminary studies, we found that the yellow embryogenic structures (YESs) and white embryogenic structures (WESs) were formed from leaf explants of *D. genkwa* when they were incubated on medium containing auxins. The embryogenic calluses (ECs) were formed from the YES after subsequent rounds of subculture on MS medium supplemented with 1 mg·L^−1^ 2,4-D. To investigate whether the embryogenic calluses and embryogenic structures from leaf explants have different potentials for plant regeneration, the EC, YES and WES were transferred onto MS medium supplemented with or without 0.1 mg·L^−1^ BA, respectively. Each treatment consisted of 12 explants in a plastic Petri dish (90 mm × 15 mm) and was repeated three times. After 4 weeks of incubation in the light, the frequency of somatic embryo development and the total number of somatic embryos from these three types of structures were determined. After a further 4 weeks of incubation in the light with subculture on the same medium, the frequency of shoot differentiation and the total number of elongated shoots from these three types of structures were determined.

### 4.4. Rooting and Acclimatization of Regenerated Plantlets

For whole plant regeneration, regenerated shoots from somatic embryos were transferred onto 1/2 MS medium containing 0.1 mg·L^−1^ IBA. The cultures were kept at 25 °C in the light (approximately 45 μmol m^−2^ s^−1^ from cool-white, fluorescent lamps with a 16 h photoperiod). Each treatment consisted of 10 explants in a plastic Petri dish (90 mm × 15 mm) and was repeated three times. After carefully removing the agar, rooted plantlets were washed with running water and transferred to potting soil. After sealing with wrap to maintain humidity, the plants were grown under light culture conditions for 10 days. When new leaves emerged from the plant, the wrap was removed and the plants were grown in a greenhouse.

### 4.5. Statistical Analysis

Statistical analysis between different groups was evaluated with *t*-test. At least three biological replicates were performed for each analysis. Quantitative data are expressed as mean ± standard deviation (SD). Student’s *t*-test was conducted in Excel.

## 5. Conclusions

In conclusion, an efficient plant regeneration system from leaf-derived embryogenic structure cultures of *D. genkwa* was established in this study. These systems can be applied to mass proliferation and genetic modification for pharmaceutical metabolite production in *D. genkwa.* In addition, we are going to investigate the possibility of the mass production of somatic embryos through cell suspension cultures for the production of useful secondary metabolites.

## Data Availability

The data presented are contained within the article or Supplementary Materials.

## Supplementary Materials

The following supporting information can be downloaded at: https://www.mdpi.com/article/10.3390/plants12112175/s1, Figure S1: Somatic embryogenesis from leaf explant cultures of *D. genkwa* cultured on MS medium containing IBA; Figure S2: Effect of concentration of growth regulators on embryogenic structure formation from leaf explant cultures of *D. genkwa* in MS (A) and WPM (B) media after 8 weeks culture in light condition; Figure S3: Effect of BA concentration on direct shoot formation from leaf explant cultures of *D. genkwa* in MS and WPM media with or without light after 8 weeks cultures.

## References

[1] Halda J.J. (1998). Some taxonomic problems in the genus *Daphne* L.. Acta Mus. Richnoviensis Sect. Nat..

[2] Moshiashvili G., Tabatadze N., Mshvildadze V. (2020). The genus *Daphne*: A review of its traditional uses, phytochemistry and pharmacology. Fitoterapia.

[3] Lee M.-Y., Park B.-Y., Kwon O.-K., Yuk J.-E., Oh S.-R., Kim H.-S., Lee H.-K., Ahn K.-S. (2009). Anti-inflammatory activity of (−)-aptosimon isolated from *Daphne genkwa* in RAW264. 7 cells. Int. Immunopharmacol..

[4] Li S., Chou G., Hseu Y., Yang H., Kwan H., Yu Z. (2013). Isolation of anticancer constituents from flos genkwa (*Daphne genkwa* Sieb. et Zucc.) through bioassay-guided procedures. Chem. Cent. J..

[5] Park B.-Y., Min B.-S., Oh S.-R., Kim J.-H., Bae K.-H., Lee H.-K. (2006). Isolation of flavonoids, a biscoumarin and an amide from the flower buds of *Daphne genkwa* and the evaluation of their anti-complement activity. Phytother. Res..

[6] Zhou D.-C., Zheng G., Jia L.-Y., He X., Zhang C.-F., Wang C.-Z., Yuan C.-S. (2021). Comprehensive evaluation on anti-inflammatory and anti-angiogenic activities in vitro of fourteen flavonoids from *Daphne Genkwa* based on the combination of efficacy coefficient method and principal component analysis. J. Ethnopharmacol..

[7] Brickell C., White R. (2000). A quartet of new Daphnes. New Plantsman.

[8] Noshad D., Miresmaili S., Riseman A., Ekramoddoullah A. (2009). In Vitro propagation of seven *Daphne* L. species. Plant Cell Tissue Organ Cult..

[9] Marks T.R., Simpson S.E. (2000). Interaction of explant type and indole-3-butyric acid during rooting in vitro in a range of difficult and easy-to-root woody plants. Plant Cell Tissue Organ Cult..

[10] Noshad D., Riseman A., Punja Z. (2007). Evaluation of *Daphne* germplasm for resistance to *Daphne* sudden death syndrome caused by the soil-borne pathogen *Thielaviopsis basicola*. HortScience.

[11] Moran J. (1995). Daphne viral diseases. Agric. Notes.

[12] Moraes R.M., Cerdeira A.L., Lourenço M.V. (2021). Using micropropagation to develop medicinal plants into crops. Molecules.

[13] Hanus-Fajerska E., Wiszniewska A., Czaicki P. (2012). Effectiveness of *Daphne* L. (Thymelaeaceae) in vitro propagation, rooting of microshoots and acclimatization of plants. Acta Agrobot..

[14] Wiszniewska A., Hanus-Fajerska E., Grabski K., Tukaj Z. (2013). Promoting effects of organic medium supplements on the micropropagation of promising ornamental *Daphne species* (Thymelaeaceae). In Vitro Cell. Dev. Biol.-Plant.

[15] Nowakowska K., Pacholczak A., Tepper W. (2019). The effect of selected growth regulators and culture media on regeneration of *Daphne mezereum* L. ‘Alba’. Rend. Lincei Sci. Fis. Nat..

[16] Nowakowska K., Pacholczak A. (2020). Comparison of the effect of meta-topolin and benzyladenine during *Daphne mezereum* L. micropropagation. Agronomy.

[17] Murashige T., Skoog F. (1962). A revised medium for rapid growth and bio assays with tobacco tissue cultures. Physiol. Plant.

[18] Lloyd G., McCown B. (1980). Commercially-feasible micropropagation of mountain laurel, Kalmia latifolia, by use of shoot-tip culture. Comb. Proc. Int. Plant Propag. Soc..

[19] Giri C., Shyamkumar B., Anjaneyulu C. (2004). Progress in tissue culture, genetic transformation and applications of biotechnology to trees: An overview. Trees.

[20] Isah T. (2016). Induction of somatic embryogenesis in woody plants. Acta Physiol. Plant.

[21] Guan Y., Li S.-G., Fan X.-F., Su Z.-H. (2016). Application of somatic embryogenesis in woody plants. Front. Plant Sci..

[22] Gaj M.D. (2004). Factors influencing somatic embryogenesis induction and plant regeneration with particular reference to *Arabidopsis thaliana* (L.) Heynh. Plant Growth Regul..

[23] Zhang M., Wang A., Qin M., Qin X., Yang S., Su S., Sun Y., Zhang L. (2021). Direct and indirect somatic embryogenesis induction in *Camellia oleifera* Abel. Front. Plant Sci..

[24] Zou S., Yao X., Zhong C., Li D., Wang Z., Huang H. (2019). Recurrent somatic embryogenesis and development of somatic embryos in *Akebia trifoliata* (Thunb.) Koidz (Lardizabalaceae). Plant Cell Tissue Organ Cult..

[25] Kuo H.-L., Chen J.-T., Chang W.-C. (2005). Efficient plant regeneration through direct somatic embryogenesis from leaf explants of *Phalaenopsis* ‘Little Steve’. In Vitro Cell. Dev. Biol.-Plant.

[26] Zhao J., Cui J., Liu J., Liao F., Henny R.J., Chen J. (2012). Direct somatic embryogenesis from leaf and petiole explants of *Spathiphyllum* ‘Supreme’ and analysis of regenerants using flow cytometry. Plant Cell Tissue Organ Cult..

[27] Ramakrishna D., Shasthree T. (2016). High efficient somatic embryogenesis development from leaf cultures of *Citrullus colocynthis* (L.) Schrad for generating true type clones. Physiol. Mol. Biol. Plants.

[28] Venkataiah P., Bhanuprakash P., Kalyan S.S., Subhash K. (2016). Somatic embryogenesis and plant regeneration of *Capsicum baccatum* L.. J. Genet. Eng. Biotechnol..

[29] Shen H.-J., Chen J.-T., Chung H.-H., Chang W.-C. (2018). Plant regeneration via direct somatic embryogenesis from leaf explants of *Tolumnia* Louise Elmore ‘Elsa’. Bot. Stud..

[30] Liang H., Xiong Y., Guo B., Yan H., Jian S., Ren H., Zhang X., Li Y., Zeng S., Wu K. (2020). Shoot organogenesis and somatic embryogenesis from leaf and root explants of *Scaevola sericea*. Sci. Rep..

[31] Xiong Y., Chen S., Wu T., Wu K., Li Y., Zhang X., da Silva J.A.T., Zeng S., Ma G. (2022). Shoot organogenesis and somatic embryogenesis from leaf and petiole explants of endangered *Euryodendron excelsum*. Sci. Rep..

[32] Phillips G.C., Garda M. (2019). Plant tissue culture media and practices: An overview. In Vitro Cell. Dev. Biol.-Plant.

[33] Zhang Y., Cao Y., Wang Y., Cai X. (2023). Somatic embryogenesis induction and genetic stability assessment of plants regenerated from immature seeds of *Akebia trifoliate* (Thunb.) Koidz. Forests.

[34] Thorpe T.A., Stasolla C., Bhojwani S.S., Soh W.Y. (2001). Somatic embryogenesis. Current Trends in the Embryology of Angiosperms.

[35] Raghavan V. (2004). Role of 2,4-dichlorophenoxyacetic acid (2,4-D) in somatic embryogenesis on cultured zygotic embryos of *Arabidopsis*: Cell expansion, cell cycling, and morphogenesis during continuous exposure of embryos to 2,4-D. Am. J. Bot..

[36] Nic-Can G.I., Loyola-Vargas V.M., Loyola-Vargas V.M., Ochoa-Alejo N. (2016). The role of the auxins during somatic embryogenesis. Somatic Embryogenesis: Fundamental Aspects and Applications.

[37] Wójcik A.M., Wójcikowska B., Gaj M.D. (2020). Current perspectives on the auxin-mediated genetic network that controls the induction of somatic embryogenesis in plants. Int. J. Mol. Sci..

[38] Li M., Wrobel-Marek J., Heidmann I., Horstman A., Chen B., Reis R., Angenent G.C., Boutilier K. (2022). Auxin biosynthesis maintains embryo identity and growth during BABY BOOM-induced somatic embryogenesis. Plant Physiol..

[39] Karami O., Philipsen C., Rahimi A., Nurillah A.R., Boutilier K., Offringa R. (2023). Endogenous auxin maintains embryonic cell identity and promotes somatic embryo development in *Arabidopsis*. Plant J..

[40] Hazubska-Przybył T., Ratajczak E., Obarska A., Pers-Kamczyc E. (2020). Different roles of auxins in somatic embryogenesis efficiency in two *Picea* species. Int. J. Mol. Sci..

[41] Michler C.H., Lineberger R.D. (1987). Effects of light on somatic embryo development and abscisic levels in carrot suspension cultures. Plant Cell Tissue Organ Cult..

[42] Kintzios S.E., Taravira N. (1997). Effect of genotype and light intensity on somatic embryogenesis and plant regeneration in melon (*Cucumis melo* L.). Plant Breed..

[43] Chan A., Stasolla C. (2023). Light induction of somatic embryogenesis in Arabidopsis is regulated by PHYTOCHROME E. Plant Physiol. Biochem..

[44] Armstrong C., Green C. (1985). Establishment and maintenance of friable, embryogenic maize callus and the involvement of L-proline. Planta.

[45] Kim H.S., Zhang G., Juvik J.A., Widholm J.M. (2010). *Miscanthus* × *giganteus* plant regeneration: Effect of callus types, ages and culture methods on regeneration competence. GCB Bioenergy.

[46] Cai T., Butler L. (1990). Plant regeneration from embryogenic callus initiated from immature inflorescences of several high-tannin sorghums. Plant Cell Tissue Organ Cult..

[47] Brisibe E.A., Miyake H., Taniguchi T., Maeda E. (1994). Regulation of somatic embryogenesis in long-term callus cultures of sugarcane (*Saccharum officinarum* L.). New Phytol..

[48] Zhang X., Wang Y., Yan Y., Peng H., Long Y., Zhang Y., Jiang Z., Liu P., Zou C., Peng H. (2019). Transcriptome sequencing analysis of maize embryonic callus during early redifferentiation. BMC Genom..

