# Supplementary material for: Efficient Plant Regeneration System from Leaf Explant Cultures of Daphne genkwa via Somatic Embryogenesis
Source: Plants (Basel). 2023 May 30;12(11):2175. doi: 10.3390/plants12112175 (PMC10255457; doi:10.3390/plants12112175)
Supplement: Supplementary file 1 [file plants-12-02175-s001.zip › plants-2405572-supplementary.pdf]

Supplementary Materials

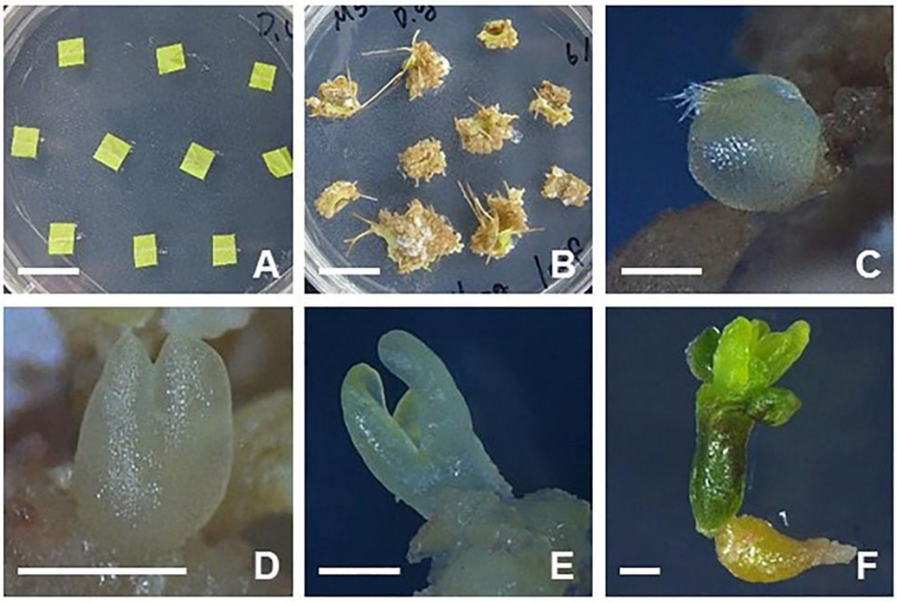

Supplementary Figure S1: Somatic embryogenesis from leaf explants cultures of *D. genkwa* cultured on Murashige and Skoog medium containing IBA. A: Leaf explants; B: Initiation of embryogenic structures formation and adventitious root formation from leaf explants; C: Globular-shaped embryo formation; D–E: Heart and torpedo-shaped somatic embryo; F: Shoots conversion from somatic embryo. Scale bars represent A, B: 1 cm; C: 1 mm; D–F: 2 mm, respectively.

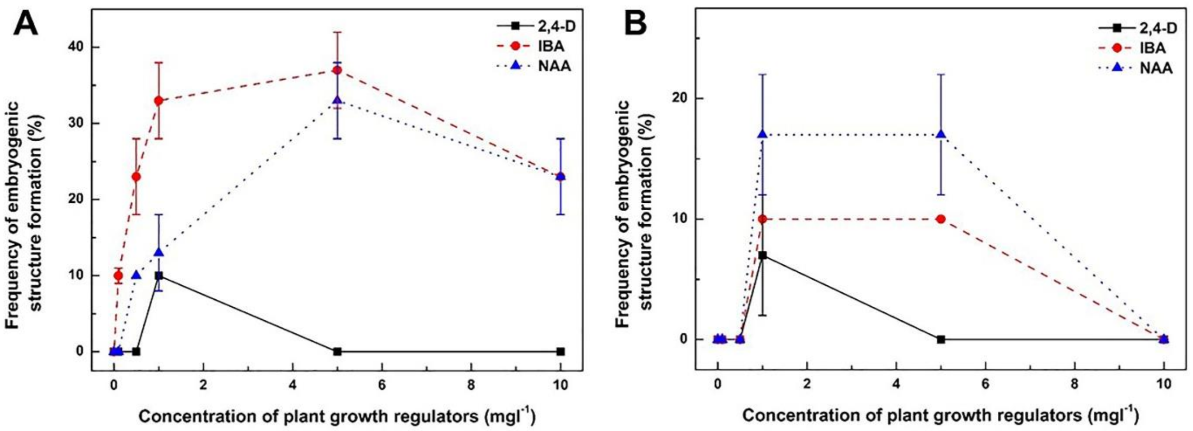

Supplementary Figure S2: Effect of concentration of growth regulators on embryogenic structure formation from leaf explants cultures of *D. genkwa* in MS (A) and WPM (B) media after 8 weeks culture in light condition.

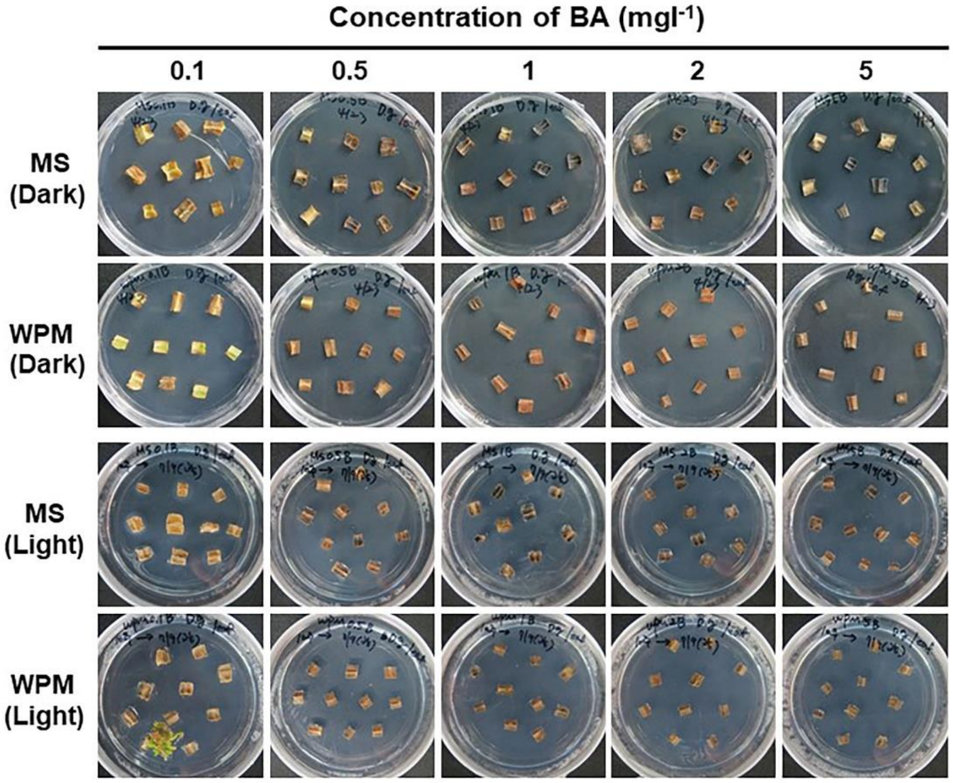

Supplementary Figure S3: Effect of BA concentration on direct shoot formation from leaf explants cultures of *D. genkwa* in MS and WPM media with or without light after 8 weeks cultures.
